# The true nature of rotary movements in rotaxanes†
†Electronic supplementary information (ESI) available: The definition of the reaction coordinates, *η*_1_ and *η*_2_, characterizing the rotations of the blocking groups. Two-dimensional maps along *η*_1_ and *η*_2_. Four conformations corresponding to four lowest minima in the previous map. Time evolution of the root-mean-square deviation over the gradients. Definition of the reaction coordinate, *φ*, characterizing the transformation of an amide bond from *syn* to *anti* conformation and free-energy profile along *φ*. Validation of CGenFF. Selection of collective variables. Rational design of molecular machines. See DOI: 10.1039/c5sc03022f


**DOI:** 10.1039/c5sc03022f

**Published:** 2015-10-13

**Authors:** Peng Liu, Xueguang Shao, Christophe Chipot, Wensheng Cai

**Affiliations:** a State Key Laboratory of Medicinal Chemical Biology (Nankai University) , Tianjin , 300071 , China; b Research Center for Analytical Sciences , College of Chemistry , Nankai University , Tianjin Key Laboratory of Biosensing and Molecular Recognition , Collaborative Innovation Center of Chemical Science and Engineering (Tianjin) , Tianjin 300071 , China . Email: wscai@nankai.edu.cn; c Laboratoire International Associé Centre National de la Recherche Scientifique et University of Illinois at Urbana–Champaign , Unité Mixte de Recherche No. 7565 , Université de Lorraine , B.P. 70239 , 54506 Vandoeuvre–lès–Nancy cedex , France; d Theoretical and Computational Biophysics Group , Beckman Institute , University of Illinois at Urbana–Champaign , Urbana , Illinois 61801 , USA . Email: chipot@ks.uiuc.edu; e Department of Physics , University of Illinois at Urbana–Champaign , Urbana , Illinois 61801 , USA

## Abstract

Disentangling the different movements observed in rotaxanes is critical to characterize their function as molecular and biological motors. How to achieve unidirectional rotation is an important question for successful construction of a highly efficient molecular motor. The motions within a rotaxane composed of a benzylic amide ring threaded on a fumaramide moiety were investigated employing atomistic molecular dynamics simulations. The free-energy profiles describing the rotational process of the ring about the thread were determined from multi-microsecond simulations. Comparing the theoretical free-energy barriers with their experimental counterpart, the *syn*–*anti* isomerization of the amide bond within the ring was ruled out. The free-energy barriers arise in fact from the disruption of hydrogen bonds between the ring and the thread. Transition path analysis reveals that complete description of the reaction coordinate requires another collective variable. The free-energy landscape spanned by the two variables characterizing the coupled rotational and shuttling processes of the ring in the rotaxane was mapped. The calculated free-energy barrier, amounting to 9.3 kcal mol^–1^, agrees well with experiment. Further analysis shows that shuttling is coupled with the isomerization of the ring, which is not limited to a simplistic chair-to-chair transition. This work provides a cogent example that contrary to chemical intuition, molecular motion can result from complex, entangled movements requiring for their accurate description careful modeling of the underlying reaction coordinate. The methodology described here can be used to evaluate the different components of the multifaceted motion in rotaxanes, and constitutes a robust tool for the rational design of molecular machines.

## Abstract 2

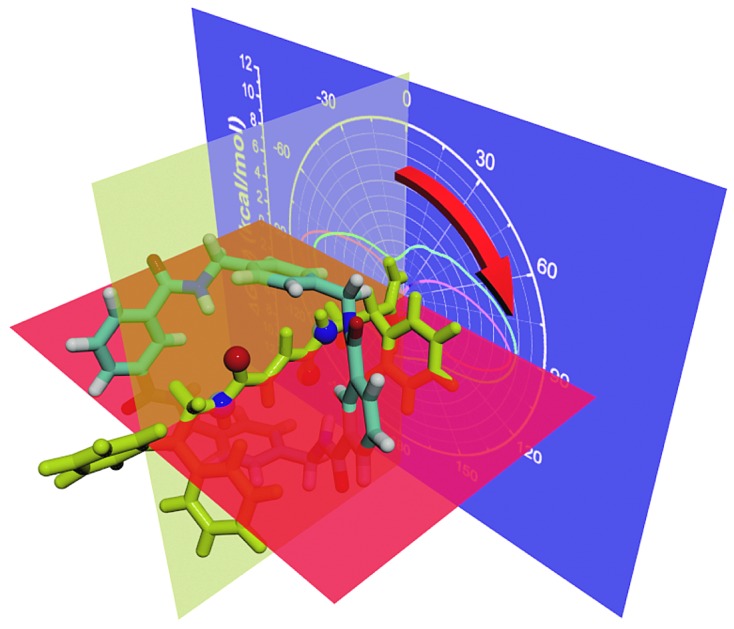
Reveal the intricate nature of movements within rotaxanes by means of multidimensional free-energy landscapes.

## Introduction

Rotaxanes,^1^ mechanically interlocked molecular architectures, are composed of a linear molecule flanked with stoppers at both termini and a macrocycle threaded onto the latter. This simple structure plays an important role as molecular switch in a variety of nanoscale objects, such as molecular memory,^2^ product lines,^3^ molecular valves,^4^ or membrane transporters,^5^ offering a wide spectrum of applications that range from chemical manufacturing to biological engineering. In these very diverse applications, the function of the rotaxane depends exquisitely upon the motion at play, notably the movement of the ring relative to the thread.^6^

In recent years, much attention has been devoted to the experimental and theoretical investigations of one kind of motion, namely shuttling of the ring across the thread. For example, shuttling of the positively charged cyclophane in the Stoddart–Heath rotaxanes confers to it the ability to interconvert between stable states with different electrical conducting properties.^7^ Due to its promising application in molecular memory, shuttling has been systematically investigated in excruciating detail by cyclic voltammetry, atom force spectroscopy,^8^ alongside quantum chemistry calculations.^9^ Meanwhile, Harada and coworkers designed a cyclodextrin-based rotaxane.^10^ The solvent-driven shuttling process was studied independently by means of NMR spectroscopy^10^ and molecular dynamics simulations.^11^ Other movements, like the rotation of the macrocycle about the thread, are, however, only sparsely documented and have been hitherto marginally examined in experimental studies.

**Fig. 1:**
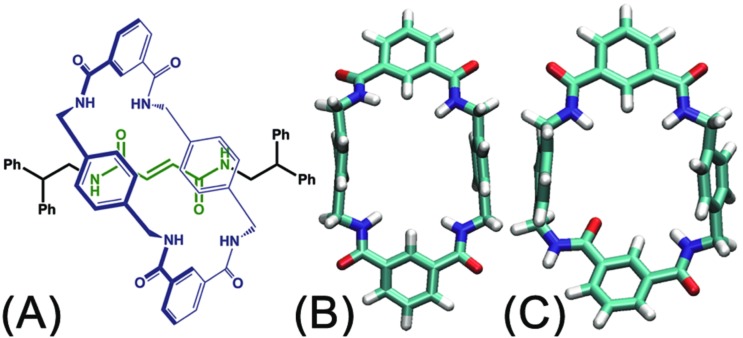
(A) Chemical structure of the rotaxane molecule studied in this contribution. (B) The ground state of the ring. All amide moieties adopt *syn* conformation. (C) The excited state of the ring. Amide moieties adopt three *syn* and one *anti* conformation.

The handful of available studies on rotary movements within rotaxanes are nearly all focused on the rotaxanes designed by Leigh and coworkers (see Fig. 1A).^12^ Within these small molecular machines, the thread composed of a fumaramide group capped with two 1,2-dibenzyl ethyl moieties is included in the cavity of a benzylic amide ring. The NH moiety of the amide groups points inwards, in the direction of the CO moiety of the fumaramide groups of the thread. The hydrogen-bonding interactions of the thread and the ring lock the rotaxanes into a stable conformation in a non-polar solvent. In the rotary movement, breaking these hydrogen bonds results in a free-energy barrier as high as *ca.* 11.5 kcal mol^–1^.^6^ Tuning the composition of the solvent,^13^ as well as the structure of the blocking groups,^15^ the conformation of the fumaramide groups^14^ affects significantly the rate of the rotation. In addition, the macrocycle undergoes a chair-to-chair conformational transition, which accompanies the precession relative to the thread. The amide moieties of the ring adopt an all-*syn* conformation (ground state) (see Fig. 1B). An alternate arrangement of the amide moieties in the ring, featuring three *syn* and one *anti* conformations (excited state), was observed in the catenane formed with the same rings (see Fig. 1C).^15^,^16^ The *syn*–*anti* isomerization of one amide moiety is hypothesized to be involved in the rotary movement. For this reason, characterizing the precession, which may be entangled with other movements of different nature, is a thorny question.

To the best of our knowledge, a complete decryption at the molecular level of the motions commonly observed in nanodevices is absent. To explore this largely unchartered research area, the rotary movement in the rotaxane designed by Leigh and coworkers (see Fig. 1A) was studied with the aid of molecular dynamics simulations combined with free-energy calculations. The free-energy profiles characterizing the rotation of the ring were determined. A detailed analysis of the structural features of the nanodevice completes the investigation to shed light on the elementary movements involved in the precession. Among these movements, shuttling is demonstrated to be highly coupled with rotation of the macrocycle by examining the free-energy changes along the latter two collective variables. The present contribution puts forth a formal theoretical framework that rests upon robust methodology as a possible avenue for *in silico* design of molecular machines.

## Simulation details

### Molecular models

The structure of the rotaxane depicted in Fig. 1, once constructed, was immersed in a box of dichloromethane. The initial size of the latter was 59.0 × 59.0 × 67.4 Å^3^ enclosing 2127 dichloromethane molecules. To improve the convergence of the free-energy calculations, the backbone of the fumaramide-based thread was restrained to remain collinear with the *z*-direction of the coordinate system by means of a harmonic potential with a force constant of 5.0 kcal mol^–1^ Å^–2^.

### Molecular dynamics simulations

All the MD simulations reported here were conducted employing the NAMD 2.10 program,^17^ with the CHARMM General Force Field (CGenFF).^18^,^19^ The validation of CGenFF can be found in the ESI.† The temperature and the pressure were maintained at 293.15 K and 1 bar, respectively, employing Langevin dynamics and the Langevin piston method.^20^ The length of the covalent bonds involving a hydrogen atom was frozen to its equilibrium value by means of the Shake/Rattle^21^ and Settle algorithms.^22^ The r-RESPA multiple-time-stepping algorithm^23^ was applied to integrate the equations of motion with a time step of 2 and 4 fs for short- and long-range interactions, respectively. Short-range van der Waals and electrostatic interactions were truncated smoothly by means of a 12 Å spherical cutoff with a switching function applied beyond 10 Å. Long-range electrostatic forces were taken into account by the particle-mesh Ewald^24^ scheme. Visualization and analysis of the MD trajectories were performed with VMD 1.9.1.^25^

### Free-energy calculations

**Fig. 2:**
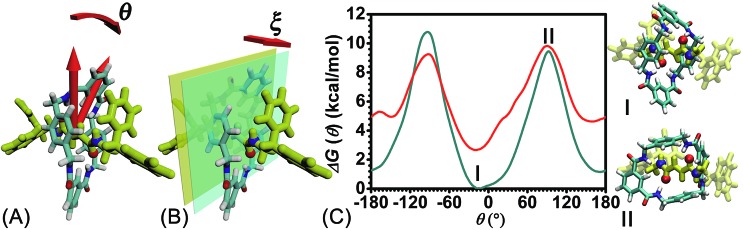
Definition of the collective variables—(A) *θ*, characterizing the rotational angle of the ring about the thread, and (B) *ξ*, the projection of the vector connecting the barycenter of the ring to the thread along the *z*-coordinate. (C) Free-energy profiles delineating the rotary movement of the ring in its ground (cyan curve) and excited states (red curve) about the thread along transition coordinate *θ*. The three-dimensional structures of configurations **I** and **II** charted on the cyan curve are shown on the right-hand side.

The free-energy landscapes reported in this contribution were generated using the ABF algorithm^26^–^30^ implemented within the Colvars module^31^ of NAMD. The model of the reaction coordinate–or transition coordinate, either one- or two-dimensional, was formed by collective variables *θ* and *ξ*, chosen as the rotational angle of the ring about the fumaramide thread and the projection of the vector connecting the center of mass of the ring to the thread along the *z*-direction, respectively (see Fig. 2A and B). Details of the selection of the collective variables are provided in the ESI.† Instantaneous values of the force were accrued in bins, either 0.1 Å or 1° × 0.1 Å wide. The variation of the free energy was determined by integrating the average force acting on *θ* and *ξ*. For configurations of the rotaxane with a ring in the ground state and in the excited state, a trajectory of 1.6 μs was generated to obtain in each case the one-dimensional free-energy profiles along *θ*. In addition, the two-dimensional free-energy map along *θ* and *ξ* and was constructed from a 1.04 μs simulation. The least free-energy pathway connecting the minima of the two-dimensional free-energy landscapes was located using the LFEP algorithm.^32^ Block average regression was applied to estimate the standard error of the free-energy change.^33^ The concept of committor^34^,^35^ was utilized to demonstrate that the model reaction coordinates formed by collective variables *θ* and *ξ* corresponds to an appropriate choice.

## Results and discussion

### Free-energy profiles

To rule out the effect of the motion of the blocking groups, the free-energy landscape characterizing the rotation of the two blocking groups was mapped (see Fig. S1 in the ESI†). Nine local minima were identified and fall into two classes (1 and 2) in terms of their free energies. Conformations pertaining to class 1 are more stable than those of class 2. The free-energy difference between local minima in each class is negligible. The rotation angles of the blocking groups were restrained to one minimum in class 1 in the subsequent calculations.

The free-energy profiles characterizing the rotary movement of the ring about the thread, in the ground and in the excited states, are depicted in Fig. 2C. Each profile possesses a wave-like shape. As can be seen, the two curves are essentially symmetrical with respect to *θ* = 0°. Two valleys found around 0° and 180° are separated by two high-energy barriers peaked around –90° and +90°. The valleys correspond to stable states wherein the fumaramide moiety of the thread is sandwiched between the *p*-xylene amines of the ring (**I**). The barriers reflect the metastable state of the rotaxane, wherein the fumaramide moiety is perpendicular to the *p*-xylene amines of the ring (**II**). Two representative configurations are gathered in Fig. 2.

The free-energy difference between the lowest points in the one-dimensional profiles was computed to be equal to 2.7 kcal mol^–1^ (see the ESI†). For the free-energy profile characterizing the rotary movement of the ring in its ground state (cyan curve), the difference between the peaks and the global minimum is estimated to be equal to 10.7 and 9.5 kcal mol^–1^, respectively. Noteworthily, the mean value, equal to 10.1 kcal mol^–1^, matches the experimental measurement. For the red curve depicting the rotation of the ring in its excited state, the free-energy differences between the peaks and the global minimum are equal to 7.2 and 6.6 kcal mol^–1^. The mean value, equal to 6.9 kcal mol^–1^, is lower than the experimental measurement, 11.5 kcal mol^–1^.^6^ This result implies that the ring in the ground state rotates about the thread and precludes any *syn*–*anti* conformational transition of the ring during precession.

**Fig. 3:**
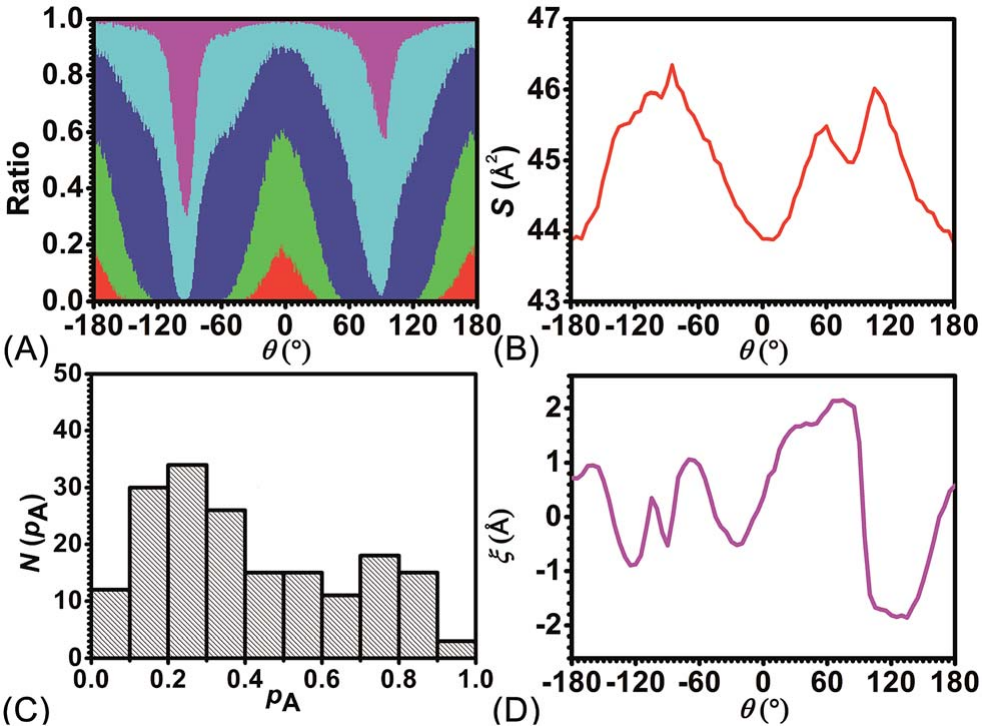
(A) The evolution of the percentage of conformations with 0-(magenta), 1-(cyan), 2-(blue), 3-(green), 4-(red) hydrogen bond(s) and (B) average area of the quadrilateral spanned by the center of the four benzyl units in the ring along the reaction coordinate, *θ*. (C) Distribution of the committor, *p*_A_, at the positions near the peak point around *θ* = 92.0°. (D) The position of the barycenter of the ring along the thread as a function of *θ*.

To delve further into the analysis of the motions at play, the evolution of the participating hydrogen bonds (HBs) was investigated. The percentage of the conformations of the rotaxane with 0, 1, 2, 3, and 4 HBs were monitored in the course of rotation as shown in Fig. 3A. In the region near 0° and 180°, corresponding to the stable states, there is at least one hydrogen bond formed between the thread and the ring. In all the conformations, the ones involving three and four HBs occupy *ca.* 60%. In the region near –90° and 90°, corresponding to the metastable state, the number of HBs formed between the ring and the thread is no more than one. Conformations with 0 HB occupy 40%. The variation of the percentage of conformations with 2, 3, 4 HBs follows the same trend observed in the free-energy profile. The percentage of conformations with no HB follows the opposite trend.

To appraise the van der Waals interaction of the ring and the thread, the average area of the quadrilateral spanned by the four benzyl units of the ring was measured (Fig. 3B). The variation of the area shares common features with the free-energy profile. However, the relative fluctuation of the area is not more than 6%. It indicates that the van der Waals interaction of the ring and the thread plays a minor role in the rotary movement. This result rationalizes the fact that the transformation of the ring from the ground state to the excited state is not required during the rotation.

To ascertain the relevance of transition coordinate *θ* used herein, a committor analysis was undertaken. Distribution of the committor, *p*_A_, at the position near the free-energy maximum (around *θ* = 92.0°) was monitored (see Fig. 3C). This distribution is pathologically widespread and clearly not Gaussian-like. It follows that collective variable *θ* is not sufficient to describe the rotary movement. Other collective variables, necessarily coupled with *θ*, ought to be introduced. In an analysis of the MD trajectory, the average position about the barycenter of the ring relative to the thread was determined (see Fig. 3D). A translational shift of the ring along the thread was observed. Evidently, a different kind of motion, namely shuttling, is involved in the rotary movement.

**Fig. 4:**
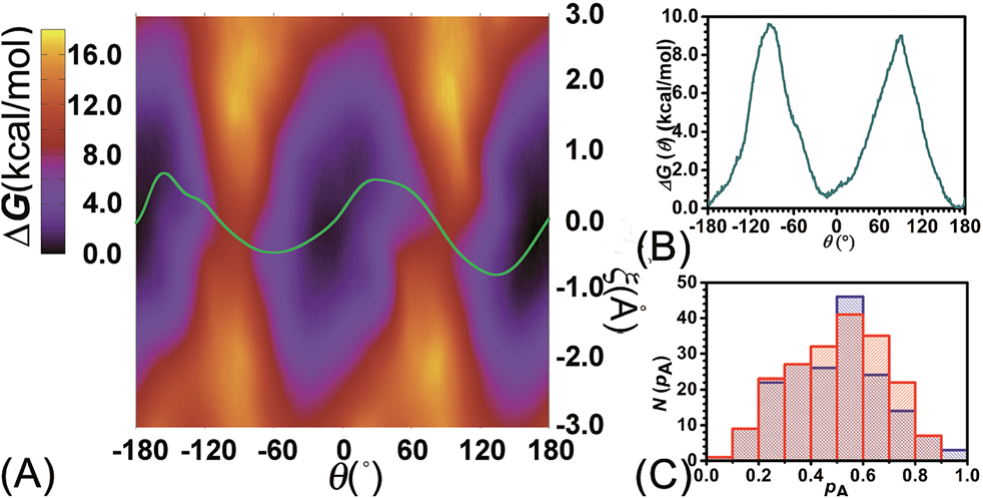
(A) Free-energy surface characterizing the concomitant rotational and shuttling processes of the rotaxane. The green line represents the least free-energy path. (B) Projection of the least free-energy path along *θ*. (C) Distribution of the committor, *p*_A_, at the positions near the saddle points around *θ* = –93.0, *ξ* = –0.25 (red) and around *θ* = 92.0, *ξ* = –0.35 (blue) in the free-energy surface.

To examine the effects of shuttling on the rotary movement, a two-dimensional free-energy map was generated along *θ* and *ξ* (see Fig. 4A). This map features two basins separated by two ridges. One gap was found on each ridge. In addition, the least free-energy path (see Fig. 4B) closely agrees with the one-dimensional free-energy profile (Fig. 1C, cyan curve). The difference between the two peaks and the global minimum is estimated to be 9.6 and 9.0 kcal mol^–1^, respectively. The mean value of these two free-energy differences, equal to 9.3 kcal mol^–1^, matches well the experimental measurement.

Relevance of the collective variables, *θ* and *ξ*, to form the transition coordinate used herein was ascertained by undertaking a committor analysis. Distributions of the committor, *p*_A_, at the position near the free-energy maxima, defined by (*θ*, *ξ*) near the saddle points, *i.e.*, (–93.0, –0.25) and (92.0, –0.35), are shown in Fig. 4C. The distributions are Gaussian-like and peaked at a value of the probability equal to 0.5. The transition coordinate formed by *θ* and *ξ*, therefore, represents a reasonable combination of collective variables to describe the rotary movement. Our results further indicate that the translational shift of the ring along the thread, that is its shuttling, constitutes an important contribution to the overall movement.

**Fig. 5:**
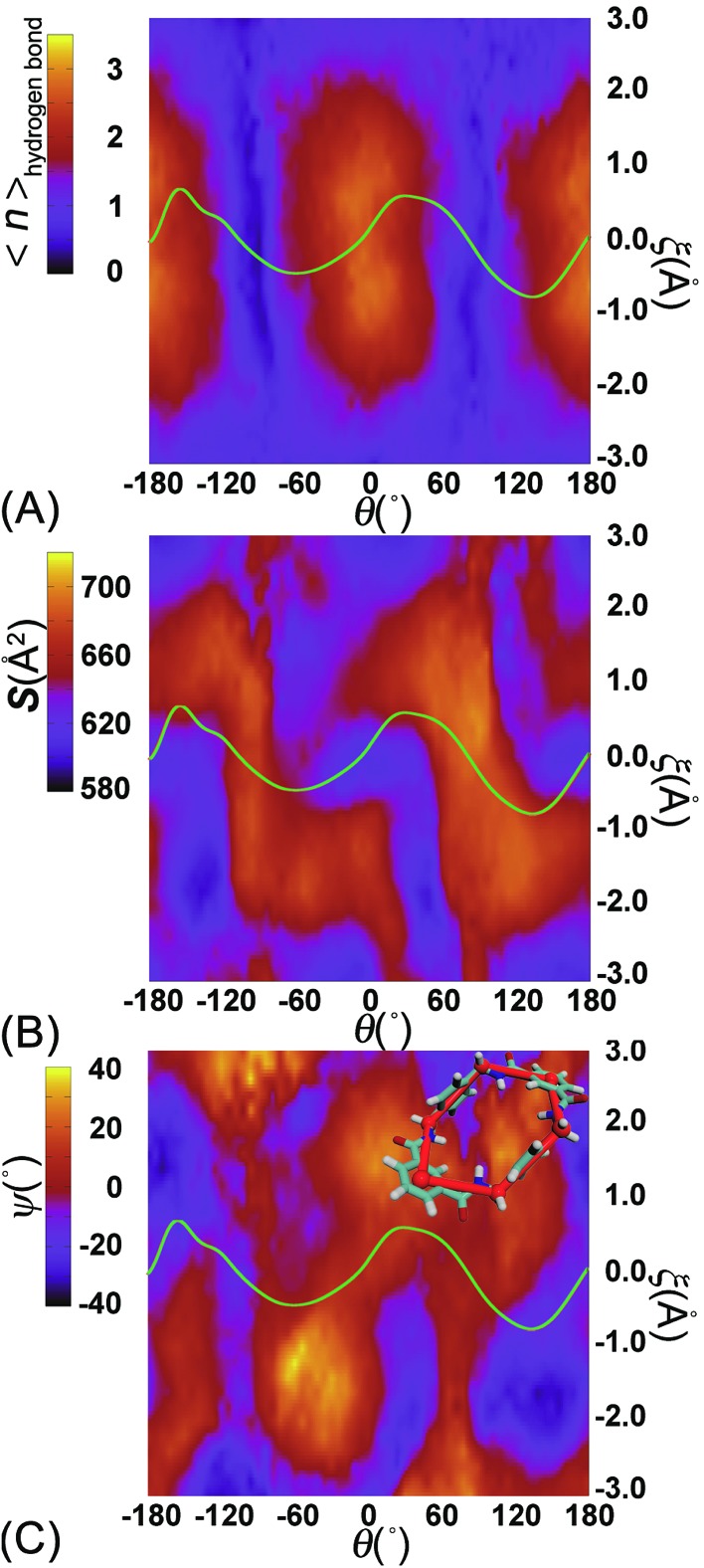
Variation of (A) the average number of hydrogen bonds and (B) the contact area between the thread and the ring, (C) the value of *ψ*, characterizing the conformation of the ring, in the space spanned by *θ* and *ξ*. The green curve is the least free-energy path located in the free-energy map. Upper corner of (C) the six groups of atoms defining the conformation of the ring.

To delve further into the role played by the translational shift of the ring, the average number of HBs (controlled by electrostatic interactions) and the area of the contact surface (controlled by van der Waals interactions) between the ring and the thread were measured and are shown in Fig. 5A and B, respectively. In Fig. 5A, the average number of HBs features two plateaus separated by two channels. Each channel is located around the ridges of the free-energy landscape and aligns with the *ξ* axis characterizing the shuttling of the ring along the thread. In the region delimited by –1 Å ≤ *ξ* ≤ 1 Å, the variation of the number of HBs is insensitive to the shuttling of the ring. The variation of the contact area between the ring and the thread shows contrasted features. Two basins defined by (*θ*, *ξ*) in the (0°, 0 Å) and (180°, 0 Å) ranges are flanked by two Z-shape ridges. A large contact area reflects a strong clash between the ring and the thread. The least free-energy path crosses the two basins and follows the same trend as the Z-shape ridges. These results indicate that shuttling of the ring is primarily caused by the clash of the ring and the thread.

To appreciate the variation of the contact area, the conformational changes of the ring were analyzed using geometric criteria defined as a function of the dihedral angles of the ring,*ψ* = (*τ*_1_ – *τ*_2_ + *τ*_3_ – *τ*_4_ + *τ*_5_ – *τ*_6_)/6where *τ*_i_ is the internal ring dihedral angle.^36^,^37^ Using this coordinate, two isomeric forms of the chair conformation correspond to values of approximately –60 and +60°. The two isomeric forms of the boat and the half-chair conformations correspond, respectively, to –30 and +30°. The twist-boat intermediate corresponds to a *ψ* value of 0. The relevant results are gathered in Fig. 5C. Two basins defined by (*θ*, *ξ*) in the (–180°, 2 Å) and (150°, –2 Å) regions correspond to an average value of *ψ* equal to –30°. Two peaks defined by (*θ*, *ξ*) in the (0°, –2 Å) and (0°, 2 Å) regions correspond to a value of *ψ* equal to 30°. The population of chair conformations is marginal. A channel and a ridge connect the two basins and the two peaks, respectively. The least free-energy path only grazes the peaks and basins and passes through the ridge and the channel. This results means that there is an obvious isomerization of the ring, which is not limited to a simplistic chair-to-chair transition.

## Conclusion

In this contribution, the rotatory movement of a rotaxane designed by Leigh and coworkers^6^ was investigated. We characterized in multi-microsecond biased simulations the rotation of the ring about the thread. This motion is not independent and another coupled motion ought to be considered. The free-energy landscape underlying the concomitant shuttling and rotation of the ring was mapped. The chosen collective variables are germane for the complete description of the movements within the rotaxane. Given a proper choice of collective variables and a suitable importance-sampling method, we were able to reproduce the data of Leigh *et al.*

The present results open an exciting perspective for the rational design of molecular machines. A lead molecular machine can be designed *de novo* by connecting components from fragment libraries. Free-energy calculations along carefully selected coarse variables can then be performed to dissect the movements at play. Certain components of the lead machine are in all likelihood suboptimal, but their replacement *in silico* by alternate, better suited fragments, followed by well-designed free-energy calculations are envisioned to be key to obtain the desired movements and speed. Ultimately, synthesis and chemical analysis will only be carried out on the most promising candidates, thereby reducing dramatically the effective cost of the new molecular machine. This strategy allows the effects of structural modifications in a molecular machine to be broadly explored, obviating the need for immediate synthesis. It also allows external conditions, such as pH, temperature and solvent nature to be optimized. A more comprehensive discussion of rational design of molecular machines is provided in the ESI.†

## Supplementary Material

Supplementary information
