# Supplementary material for: The true nature of rotary movements in rotaxanes
Source: Chem Sci. 2015 Oct 13;7(1):457–62. doi: 10.1039/c5sc03022f (PMC6090524; doi:10.1039/c5sc03022f)
Supplement: Supplementary file 1 [file SC-007-C5SC03022F-s001.pdf]

Electronic Supplementary Material (ESI) for Chemical Science.
This journal is © The Royal Society of Chemistry 2015

Electronic Supplementary Information for:

The True Nature of Rotary Movements in Rotaxanes

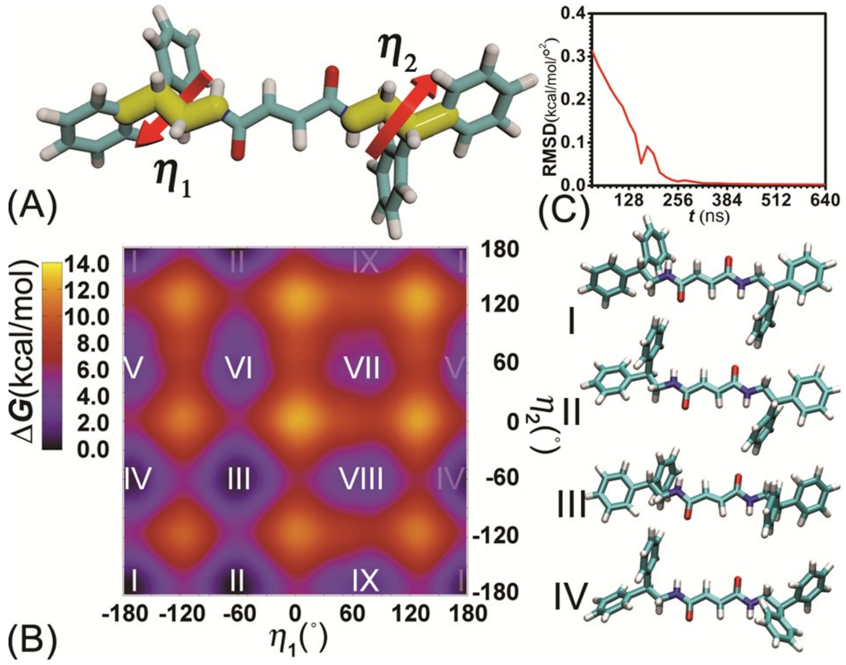

Figure S1. (A) Definition of the coarse variables, η_1 and η_2, in the rotation of the blocking groups. Rotation of the ring was suppressed. (B) Free−energy surface characterizing the rotation of the blocking groups. Nine local minima identified and successively labeled from I to IX fall into two classes in terms of their ΔG, namely, class 1: I-IV, and class 2: V-IX. The conformations corresponding to the four lowest minima, I, II, III and IV, are shown in the right panel. (C) Time-evolution of the root mean−square deviation over the gradients of the free−energy surface.

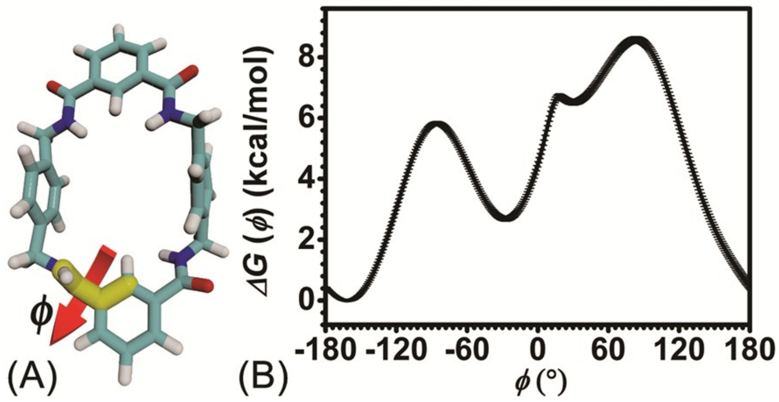

Figure S2. (A) Definition of the transition coordinate, ϕ, characterizing the rotation around the bond connecting an amide moiety to an aryl moiety (transformation of one amide moiety from its syn to its anti conformation). (B) Free−energy profile characterizing the transformation of one amide moiety.

Validation of CGenFF

The directionality of the hydrogen bonds in the rotaxane can be ascribed to the interaction of the amide moieties within the ring with the fumaramide moiety of the thread. To test the accuracy of CGenFF, the interaction of CO and NH of two amide moieties was evaluated at the QM and MM levels. The potential energy surface of two amide moieties along the vector that connects the oxygen and the nitrogen atom of each monomer at the MP2/6-31G(d) level.[1] The QM calculation was carried out using ORAC.[2] The MM calculation was performed employing NAMD with CGenFF. As shown in Figure S3, the two curves match well in the region of the global minimum. This result suggests that CGenFF can describe quantitatively the interaction involving hydrogen bonds around the most stable conformation.

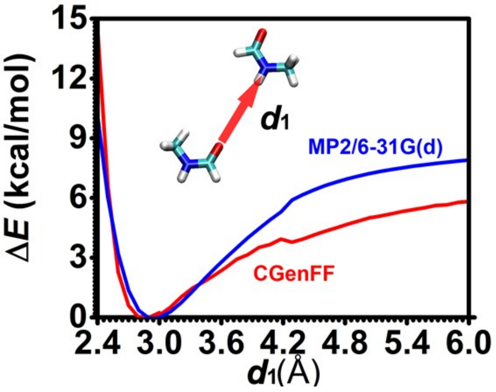

Figure S3. Potential energy surface of an amide dimer along the distance between O and N atoms.

For the conjugated portions of the rotaxane, the interactions at play can be subdivided into two groups, namely the interactions of the conjugated portion with the non-conjugated portion, and the interactions of conjugated portions. The former and latter are composed of CH-π and π-π interactions, respectively. To probe the ability of CGenFF to describe CH-π interactions, a molecular model containing one methane and one benzene molecule was built. The potential energy surface characterizing the approach of the two molecules was determined from QM and MM calculations. The QM calculation was performed at the MP2/cc-pVTZ level.[3] From Figure S4, the resulting two curves have a similar shape. However, the position of the global minimum is not consistent between the QM and the MM profiles. This result indicates that the force field is semi-quantitatively correct in describing CH-π interactions, in agreement with the conclusion of Sherrill et al.[4]

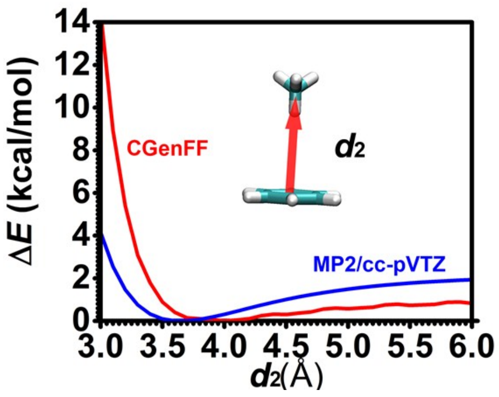

Figure S4. Potential energy surface for the methane-benzene complex.

In principle, π-π stacking is the result of a subtle balance between dispersive forces and repulsive quadrupole-quadrupole interactions. In empirical force fields, description of stacking interactions relies on the ability of minimalist intermolecular potentials based on a charge-charge Columbic term supplemented by a Lennard-Jones term to reproduce this subtle balance. Van der Waals parameters are optimized to fit experimental data, like heats of vaporization and molecular volumes.[1] Application to toy-systems, e.g., the benzene dimer, yields satisfactory result.[5] Yet, to test CGenFF, the potential energy surface characterizing the approach of benzene towards fumaramide was determined at the QM and MM levels of theory. The QM calculation was performed at df-scs-MP2/aug-cc-pVTZ.[6] The results

are gathered in Figure S5. The two curves clearly do not match, indicating that the empirical force field is not quantitatively reliable.[4] Although empirical force fields are only qualitatively correct, they have been successfully applied to investigate the binding of DNA to carbon nanotubes,[7,8] where π-π interactions are crucial.

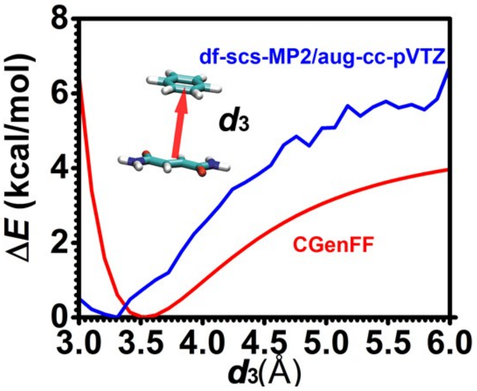

Figure S5. Potential energy surface for the benzene-fumaramide complex.

Comparing with QM, the CGenFF results are quantitatively correct for the description of the interaction involving directional hydrogen bonds around the most stable conformation, albeit only qualitatively so for the description of π-π and CH-π interactions. It is, however, fair to acknowledge that the number of options for modeling naon-objects involving π-π and CH-π interactions is rather limited. Besides, the kind of movements simulated in the present investigation are sufficiently robust that they should be only mildly affected by force-field inaccuracies. However, it will not change the overall picture of the study. Put together, we feel that CGenFF still constitutes a reasonable choice for this work.

Selection of Collective Variables

Selection of relevant collective variables to model the reaction coordinate constitutes a significant challenge. In many cases, the essence of the complex motions within molecular machines cannot be captured in a simple one-, or two-dimensional free-energy landscape, which often stems from the slow relaxation of certain degrees of freedom, reflected in hidden free-energy barriers. While one can

increase the dimensionality of the representation of the reaction coordinate, an appropriate selection of relevant collective variables is still necessary to improve the convergence of the free-energy calculation. Besides, while the choice of the model reaction coordinate does not affect the thermodynamics of the phenomena at play, it will acutely impact its kinetics.

Increasing the dimensionality of the free-energy landscape generally improves the convergence of the simulation. This improvement is perceptible whenever timescale separation is possible and a time-dependent bias acts on a collective variable associated to a slow degree of freedom. Increasing dimensionality of the model reaction coordinate, however, has stringent limitations. For obvious practical reasons, in methods like ABF, the dimensionality cannot go much beyond than three. One reasonable solution to describe the transition coordinate that underlies complex processes is transition path theory.[9,10] From the transition path sought in a multidimensional collective-variable space one can project this path in a much lower dimensional space, along which a free-energy change can be estimated. Much computational effort is generally required to determine the transition path. The choice of the coarse variables in which this path is sought also constitutes a crucial aspect in these calculations.

Rotation of the ring around the thread is the most characteristic motion of the rotaxane examined in our manuscript.[11] Chair-chair flipping of the ring was found to play an important role in the rotation process of the ring.[12] Syn-anti isomerization of the amide moieties within the ring was reported in another work.[13] A different intrinsic movement, namely the shuttling of the ring along the thread, can, however, not be neglected.[14] Rotation of the blocking groups was also visualized in the equilibrium simulation. Hence, five movements of different nature are involved in the motion of the rotaxane. Which motion is the most important? Which collective variable constitutes the best model of the reaction coordinate? The free-energy landscape for each variable was mapped. Determination of the relevant committor distributions proved, however, that none of the individual collective variables offered a satisfactory representation of the reaction coordinate. One-dimensional free-energy calculations may well be plagued by slow degrees of freedom in orthogonal space, with significant hidden free-energy barriers. Considering slow movement in orthogonal space, we mapped the two-

dimensional free-energy surface in the collective-variable subspace formed by the shuttling and the rotation of the ring. Next, we established the minimum free-energy path connecting the minima on this surface and determined the distribution of the committor function at the free-energy barrier to validate this path and the underlying collective-variable subspace.

Exploring free-energy landscapes that are multidimensional in nature represents a challenging task, in which selecting the relevant variables is crucial. While dedicated methods have proven useful to search for transition paths, e.g., the string method and its variants, careful validation is crucial.

Rational Design of Molecular Machines

Movements observed in molecular machines are usually diverse and complex. Under most circumstances, physical properties are determined post-facto, downstream from costly syntheses — sometimes revealing undesired features in the movements of the nano-objects. Such undesired features could be safely avoided by dint of a more efficient and rational strategy for the design of novel molecular machines endowed with specific functions, in a spirit similar to that of de novo drug design. The present contribution puts forth a formal theoretical framework that allows us to investigate nano-machines at the atomic level to reveal the intricate nature of the movements in such supramolecular devices.

Design of molecular machines with desired features and performance can start with an optimization from known structures, or by building novel architectures based on known structural fragments. Within the proposed theoretical framework, the free-energy landscape underlying the complex motions driving these nano-objects can then be mapped, from whence a meaningful reaction path is inferred and validated by means of a post-hoc committor analysis. In light of the free-energy barriers at play, the working rate of the molecular machines is estimated. By repeating the structural modifications and estimating the physical properties of the nano-objects in a self-consistent fashion, one can achieve the desired performance. The strategy of structural optimization can be considered from different perspectives. Replacing flexible chains in molecular machines by rigid moieties could reduce

the free-energy barrier encountered in the working process.[15] Tuning the percentage and positions of hydrophobic and hydrophilic moieties may greatly impact the free-energy barrier. For example, changing the hydrophobic chain to a hydrophilic one within a rotaxane immersed in DMSO significantly accelerates the shuttling process.[16] For electrostatically-controlled machines, neutralizing some of the charged moieties can attenuate the driving force in the working process.[17] By applying the theoretical framework put forth in this contribution, the influence of these structural and chemical modifications on the performance of the molecular machines can be quantified.

REFERENCES

(1) K. Vanommeslaeghe, E. Hatcher, C. Acharya, S. Kundu, S. Zhong, J. Shim, E. Darian, O. Guvench, P. Lopes, I. Vorobyov and A. D. Mackerell Jr, *J. Comput. Chem.*, 2010, **31**, 671.

(2) F. Neese. ORCA - an ab initio, Density Functional and Semiempirical program package, Version 2.7; University of Bonn: Germany, 2008.

(3) A. T. Macias and A. D. MacKerell Jr, *J. Comput. Chem.*, 2005, **26**, 1452.

(4) C. D. Sherrill, B. G. Sumpter, M. O. Sinnokrot, M. S. Marshall, E. G. Hohenstein, R. C. Walker and I. R. Gould, *J. Comput. Chem.*, 2009, **30**, 2187.

(5) C. Chipot, R. Jaffe, B. Maigret, D. A. Pearlman and P. A. Kollman, *J. Am. Chem. Soc.*, 1996, **118**, 11217.

(6) A. R. McDonald, E. J. Denning and A. D. MacKerell Jr, *J. Phys. Chem. A*, 2013, **117**, 1560.

(7) R. R. Johnson, A. T. Charlie Johnson and Michael L. Klein, *Nano Lett.*, 2008, **8**, 69.

(8) R. R. Johnson, A. Kohlmeyer, A. T. Charlie Johnson and Michael L. Klein, *Nano Lett.*, 2009, **9**, 537.

(9) P. G. Bolhuis, D. Chandler, C. Dellago and P. L. Geissler, *Annu. Rev. Phys. Chem.*, 2002, **53**, 291.

(10) A. C. Pan, D. Sezer and B. Roux, *J. Phys. Chem. B*, 2008, **112**, 3432.

(11) A. M. Rijs, N. Sändig, M. N. Blom, J. Oomens, J. S. Hannam, D. A. Leigh, F. Zerbetto and W. J. Buma, *Angew. Chem. Int. Ed.*, 2010, **49**, 3896.

(12) F. G. Gatti, S. León, J. K. Y. Wong, G. Bottari, A. Altieri, M. A. F. Morales, S. J. Teat, C. Frochot, D. A. Leigh, A. M. Brouwer and F. Zerbetto, *Proc. Natl. Acad. Sci. U S A.*, 2003, **100**, 10.

(13) F. G. Gatti, D. A. Leigh, S. A. Nepogodiev, A. M. Slawin, S. J. Teat, J. K. Wong. *J. Am. Chem. Soc.*, 2001, **123**, 5983.

(14) M. R. Panman, B. H. Bakker, D. den Uyl, E. R. Kay, D. A. Leigh, W. J. Buma, A. M. Brouwer, J. A. J. Geenevasen and S Woutersen, *Nature Chem.*, 2013, **5**, 929.

(15) P. G. Young, K. Hirose and Y. Tobe, *J. Am. Chem. Soc.*, 2014, **136**, 7899.

(16) Y. Kawaguchi and A. Harada, *Org. Lett.*, 2000, **2**, 1353.

(17) X. Bao, I. Isaacsohn, A. F. Drew and D. B. Smithrud, *J. Am. Chem. Soc.*, 2006, **128**, 12229.
